# The emerging roles of m^6^A modification in liver carcinogenesis

**DOI:** 10.7150/ijbs.50003

**Published:** 2021-01-01

**Authors:** Xue-yin Pan, Cheng Huang, Jun Li

**Affiliations:** 1 Inflammation and Immune Mediated Diseases Laboratory of Anhui Province, Anhui Institute of Innovative Drugs, School of Pharmacy, Anhui Medical University, Hefei, 230032, China.; 2 The Key Laboratory of Anti-inflammatory of Immune Medicines, Ministry of Education.; 3 Institute for Liver Diseases of Anhui Medical University.

**Keywords:** Epitranscriptome, N^6^-methyladenosine, Hepatocellular Carcinoma (HCC)

## Abstract

The 'epitranscriptome', a collective term for chemical modifications that influence the structure, metabolism, and functions of RNA, has recently emerged as vitally important for the regulation of gene expression. N^6^-methyladenosine (m^6^A), the most prevalent mammalian mRNA internal modification, has been demonstrated to have a pivotal role in almost all vital bioprocesses, such as stem cell self-renewal and differentiation, heat shock or DNA damage response, tissue development, and maternal-to-zygotic transition. Hepatocellular carcinoma (HCC) is prevalent worldwide with high morbidity and mortality because of late diagnosis at an advanced stage and lack of effective treatment strategies. Epigenetic modifications including DNA methylation and histone modification have been demonstrated to be crucial for liver carcinogenesis. However, the role and underlying molecular mechanism of m^6^A in liver carcinogenesis are mostly unknown. In this review, we summarize recent advances in the m^6^A region and how these new findings remodel our understanding of m^6^A regulation of gene expression. We also describe the influence of m^6^A modification on liver carcinoma and lipid metabolism to instigate further investigations of the role of m^6^A in liver biological diseases and its potential application in the development of therapeutic strategies.

## Introduction

To date, more than 170 types of RNA modifications have been identified, including 5' cap modification, poly(A) tail, pseudouridine (Ψ), N^1^-methyladenosine (m^1^A) and N^6^,2'-O-dimethyladenosine (m^6^A_m_), and N^6^-methyladenosine (m^6^A) 1. Among these modifications, m^6^A is the most abundant internal RNA modification in eukaryotic cells that widely occurs in mRNA 2 and non-coding RNAs (ncRNAs) 3-11. Numerous studies have provided evidence that m^6^A is involved in fundamental physiological and pathological RNA metabolic processes, including splicing, nuclear export, stability, translation, and decay 12-16. These studies advanced our understanding of m^6^A function and regulatory mechanisms in various human diseases.

m^6^A is highly conserved among eukaryotic species ranging from yeast 17,18, plants 19, and drosophila viruses 20,21 to mammals 22,23. However, the m^6^A filed did not progress for several decades until the availability of MeRIP-seq or m^6^A-seq techniques 24,25. Consensus motif analyses revealed that m^6^A sites depicted as “DRACH” motif (R = G or A; H = A, C, or U; where A is converted to m^6^A) in the transcriptome is not random but occurs in coding sequences (CDS), 3'-untranslated regions (3'-UTRs), and especially in the region around the stop codon 24,25. Despite the prevalence of DRACH sequences in the transcriptome, only 1-5% of these sites are methylated *in vivo*, indicating that the DRACH motif itself is not sufficient to determine m^6^A deposition.

m^6^A modification is induced by m^6^A methyltransferase complex (composed of METTL3/METTL14/METTL16, WTAP, KIAA1429, ZC3H13, HAKAI, and RBM15/15B) 26-28, removed by m^6^A demethylases (FTO and ALKBH5) 29,30, and recognized by RNA binding proteins, including YTHDF1/2/3, YTHDC1/2, IGF2BP1/2/3, hnRNPs 14,16,31-39. m^6^A modification of U6 snRNA and structural RNA, 18 S rRNA and 28 S rRNA, the 5'cap m^6^A_m_, and U2 snRNA internal m^6^A_m_ are methylated by METTL16 10,11,40, METTL5 41, ZCCHC4 9,42 and PCIF1 43,44, respectively. Recently, METTL4 was shown to be a U2 snRNA internal m^6^A_m_ methyltransferase 45.

It has been demonstrated that m^6^A modification influences gene expression and is involved in a variety of human cancers including hepatocellular carcinoma (HCC) 46. In terms of high morbidity and mortality, HCC ranks the second highest cancer 47,48. Multiple factors can trigger or worsen the pathology of HCC, including chronic hepatitis B and C viral infection, chronic alcohol consumption, obesity, metabolic disorders, and non-alcoholic fatty liver disease (NAFLD) 49. Surgical resection is the only curative therapy and sorafenib is the only approved drug for the treatment of HCC. The survival rate is up to 70% in patients with a tumor size of less than 2 cm 50. Therefore, early diagnosis is essential for HCC treatment. Numerous studies have demonstrated an important role of epigenetic modifications including DNA and histone modification in regulating HCC progression 51. There is increasing evidence that total m^6^A levels and abnormal expression of m^6^A regulators are altered and associated with HCC clinical prognosis 52,53. Furthermore, the m^6^A modification regulators play an oncogenic or tumor suppressor role in HCC by influencing the expression of special genes 54-56. Therefore, in this review, we focused on recent progress in m^6^A detection, the function and the mechanism of m^6^A regulators in lipid metabolism, hepatic virus infection, and liver carcinogenesis, and it may provide a potential novel therapeutic and prognosis targets for HCC.

## Development of m^6^A detection approaches

The global levels of m^6^A in total RNA can be detected by liquid chromatography coupled to tandem mass spectrometry (HPLC-MS/MS), dot-blot analysis 29, and m^6^A Methylation Quantification Kit 12,29. The most commonly used approach for detecting transcriptome-wide m^6^A levels is MeRIP-seq 24 or m^6^A-seq 25 that can detect m^6^A sites in a 100-200 nt resolution. Then the SCARLET method was developed to qualify the m^6^A levels of individual modification sites 57 but it is not feasible for high-throughput applications. Subsequently, MeRIP-seq and m^6^A-seq methods were improved with an added ultraviolet crosslinking step to identify m^6^A sites at the single-nucleotide resolution termed PA-m^6^A-seq (photo-crosslinking-assisted m^6^A sequencing strategy) 58,59 or m^6^A-CLIP-seq (UV cross-linked m^6^A antibody-bound RNA sample) 60,61. Then, MAZTER-seq and m^6^A-REF-seq, were developed that relied on MazF RNase or ChpBK RNase by two independent groups 62,63. However, MATER-seq or m^6^A-REF-seq sensitive to m^6^A sites occurs only in the ACA motif context. DART-seq, which rely on the cytidine deaminase APOBEC1 refused with YTH domain contain protein was used to induce C to U deamination at sites adjacent to m^6^A residues 64. However, the quality of DART-seq relies on transfection efficiency, and its application is limited. DAPT-seq also loses some m^6^A sites and has higher false-positive and false-negative rates. Recently, an FTO-assisted m^6^A selective chemical labeling method (termed m^6^A-SEAL) was developed 65. However, the resolution of m^6^A-SEAL is about 200 nt which needs to be improved in the future. In m^6^A-label-seq method, Se-allyl-l-selenohomocysteine substituted the methyl group with allyl on SAM 66. Subsequently, cellular RNAs were modified with N^6^-allyladenosine (a^6^A) at the presumed m^6^A-generating adenosine sites and will be further pinpointed by iodination-induced misincorporation at the opposite side in the complementary DNA during reverse transcription. The rapid development of m^6^A detection method will greatly accelerate the discovery and validation of m^6^A modification sites in human genome species, which will further facilitate the development of new biomarkers for cancer prognosis and classification.

## Dynamic regulation of m^6^A

**Figure 1:**
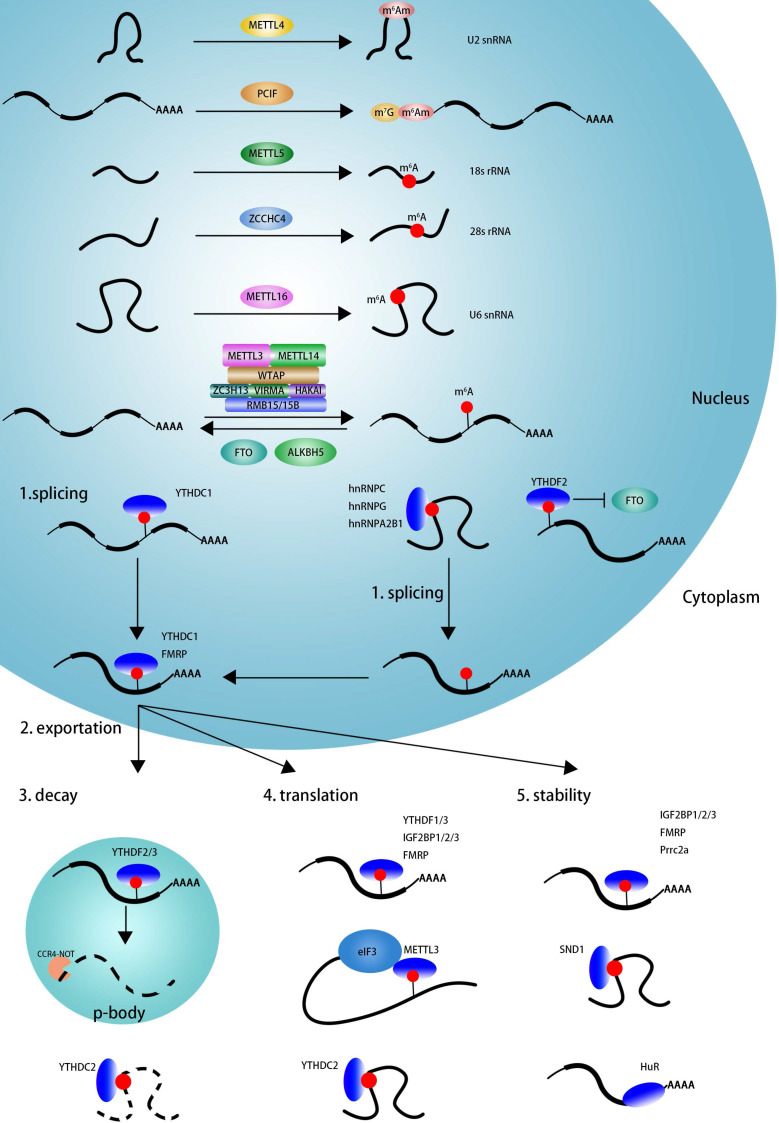
** The dynamic regulation and function of m^6^A and m^6^A_m_.** The m^6^A modification is methylated by the methyltransferase complex (MTC, also known as m^6^A 'writer'), composed of the core catalytic subunit METTL3-METTL14 and the regulator proteins WTAP, VIRMA, ZC3H13, RBM15/RBM15B and so on. METTL16 alone can methylated m^6^A deposition in structural RNA and snRNA. METTL5 is responsible for m^6^A deposition in 18 S rRNA and facilitate ZCCHC4 as 28 S rRNA methylase. METTL4 mediate internal m^6^Am modification in U2 snRNA. m^6^A modification can be eliminated by m^6^A 'eraser' (FTO and ALKBH5). The function of m^6^A is mediated by m^6^A 'reader', which affects RNA splicing, export, decay, stabilization and translation. YTHDC1 and hnRNPs (hnRNPA2B1, hnRNPC and hnRNPG) facilitate RNAs alternative splicing. The nuclear export of m^6^A containing RNAs is mediated by YTHDC1 and FMRP. The nuclear YTHDF2 binds with m^6^A in 5'UTR prevent FTO demethylation and promote cap-independent translation. The decay of m^6^A modified RNAs is mediated by YTHDF2/YTHDF3 and YTHDC2. The translation is facilitated by YTHDF1/YTHDF3, IGF2BP1/2/3, FMRP, METTL3 and YTHDC2. IGF2BP1/2/3, FMRP, PRRC2a and SND1 facilitate to keeping methylated RNAs stability.

As showed in **Figure 1**, m^6^A modification can be induced by m^6^A methyltransferase complex (MTC, also known as m^6^A 'writers') 67,68, which is composed of the core catalytic subunit METTL3/METTL14 67 and regulatory proteins, including WTAP, VIRMA, ZC3H13, HAKAI, RBM15/RBM15B 28,69. Unlike METTL3 complex that preferentially methylates single-stranded RNAs (ssRNAs), METTL16 functions alone and selectively methylates structured RNAs in the UACAGAGAA sequence 40. Recently, METTL5 is reported to catalyze m^6^A modification in 18 S rRNA at position A_1832_, and ZCCHC4 induces m^6^A sites in 28 S rRNA at position A_4220_, and METTL5 is stabilizes by TRMT112 9,41,42. The cap structure, 7-methylguanosine (m^7^G), is linked to the first transcribed nucleotide (X) that is methylated at the ribose O2 position. If the first nucleotide is adenosine, it can be further methylated on the N^6^ position, termed m^6^A_m_. m^6^A_m_ modification adjacent to the m^7^G cap is a reversible modification catalyzed by PCIF1 (CAPAM, cap-specific adenosine N^6^-methyltransferase) 43,44,70,71. METTL4 has recently been identified as a novel internal m^6^A_m_ methyltransferase responsible for N^6^-methylation of m^6^A_m30_ on U2 snRNA with a preference for the AAG motif 45.

The m^6^A mark may be removed by RNA demethylases (also known as m^6^A 'erasers'), including FTO and ALKBH5 12,29. The identification of FTO suggested that m^6^A modification is dynamic and reversible 29. Further studies reveal that FTO can remove m^6^A and m^6^A_m_ in mRNA and U6 snRNA, and m^1^A in tRNA 70,72. ALKBH5 seems to specific demethylated m^6^A modification in RNA 12.

m^6^A is involved in numerous RNA processes, including splicing, export, decay, stability, and translation and exerts its functions mainly by recruiting m^6^A-binding proteins (also known as m^6^A 'readers'). As displayed in **Figure 1**, YTHDC1, hnRNPC, hnRNPG, and hnRNPA2B1 may regulate mRNA splicing by recognizing m^6^A sites on pre-mRNA in a direct or indirect (also known as “m^6^A switch”) manner 14,33,38,73. YTHDC1 and FMRP could mediate nuclear export of m^6^A modified RNAs into cytoplasm 74-76. The nuclear YTHDF2 binds m^6^A sites in 5'UTR and prevents demethylation by FTO and thereby promotes cap-independent translation 77. In the cytoplasm, YTHDF2, YTHDF3, YTHDC2 could mediate m^6^A deposited transcripts degradation in p-bodies, whereas IGF2BP1/2/3, FMRP, Prrc2a, SND1, and HuR play an important role in maintaining m^6^A-containing transcripts stabilization 35,78-84. YTHDF1, YTHDF3, YTHDC2, IGF2BP1/2/3, FMRP, and cytoplasm METTL3 promotes m^6^A deposited mRNA translation 31,32,35,36,85-89. In summary, m^6^A influence RNA every aspect of RNA metabolism depending on the functions of various readers.

## Site specificity of m^6^A deposition

Why the distribution of m^6^A sites on DRACH motif is not random but occurs in CDS and 3'-UTR regions, especially around the stop codon, despite the high prevalence of DRACH 25? Yue *et al.* demonstrated that VIRMA can interact with WTAP-HAKAI-ZC3H13 to recruit METTL3/METTL14 and guide region-selective methylation in 3'UTR and near stop codon 28. FTO preferentially targets pre-mRNAs in intronic regions and regulates alternative splicing and 3'end processing 64. Huang *et al.* described that Histone H3 trimethylation at Lys36 (H3K36me3) can guide m^6^A modification globally 90. H3K36me3 can be recognized and bound directly by METTL14, and then facilitates the binding of MTC to adjacent RNA polymerase II, regulating m^6^A deposition in actively transcribed nascent RNAs. This study revealed a cross-talk between histone modification and m^6^A deposition. Several studies indicated that transcription factors can also influence m^6^A deposition. For instance, ZFP217 interacts with several epigenetic regulators and modulates m^6^A deposition on their transcripts by sequestering the enzyme METTL3 13. The CAATT-box binding protein CEBPZ recruits METTL3 to chromatin and promotes m^6^A modification within the coding region of the associated mRNA transcripts 91. The interaction of SMAD2/3 with METTL3/METTL14/WTAP complex mediates m^6^A modification in a subset of transcripts involved in early cell fate decisions 92. By using the CRISPR-Cas9 technology, the m^6^A modification site can be edited by fusing dCas9 (dead Cas9) with single-chain methyltransferase domains derived from METTL3 and METTL14 or full-length of m^6^A demethylase (ALKBH5 or FTO) 88. Programmable m^6^A editing can be realized by this approach providing a mechanistic understanding of epitranscriptome.

## Aberrant m^6^A regulation in liver disease

### Lipid metabolism

HCC is the second most frequent cancer in the world with high morbidity and mortality 49. Viral hepatitis, alcohol abuse, and nonalcoholic steatohepatitis (NASH) progress to HCC. Therefore, the prevention of HCC is focused on averting virus infection (hepatitis B and C viruses), inflammation, and obesity 93. NAFLD is a risk factor predisposing HCC formation and is associated with metabolic syndromes, including diabetes and obesity. Transcriptome-wide m^6^A profile has revealed that m^6^A-containing gene exhibit a high overlap in human HepG2 cell lines and mouse livers. Moreover, m^6^A-modified regions in liver tissues are enriched of single nucleotide polymorphisms (SNPs) related to lipid traits 94. Functional enrichment analysis of m^6^A-modified genes in porcine liver at three developmental stages are involved in regulating growth, development, metabolic process and protein catabolic processes 95. The diverse pattern of m^6^A-modification on genes in different stage had metabolic functions required for or specific to this stage, which indicating that the liver may be exposed to different stimuli during development. High-fat diet (HFD) induced m^6^A modification enriched in gene associated with lipid-associated processes, including cellular lipid metabolism, fatty acid metabolism, response to fatty acids and TG metabolism 96. Xie *et al.* reveals that elevated METTL3 expression in Type 2 diabetes (T2D) patients inhibits hepatic insulin sensitivity via N^6^-methylation of Fasn (fatty acid synthase) mRNA, and subsequently promotes fatty acid metabolism 97. Approximately one-tenth of liver genes have been identified as rhythmic, however only one-fifth of those genes are driven by de novo transcription, suggesting that both transcriptional and epigenetic mechanism underlie the mammalian circadian clock 98. At the core of the mammalian circadian clock gene regulatory network, the bHLH-PAS transcriptional activators BMAL1, CLOCK and NPAS2 activate the Period (Per1, Per2) and Cryptochrome (Cry1, Cry2) genes whose transcripts and proteins slowly accumulate during the daytime 98. Some circadian clock-dependent pathway involved in regulating both anabolism and catabolism, such as lipid metabolism, glucose metabolism and bile acid synthesis 99. Recently, a research showed that m^6^A modification involved in regulating this process, liver-specific deletion of Bmal1 (Bmal1^-/-^) results in high levels of ROS and elevated oxidative damage, and subsequently ROS significantly elevates METTL3 expression, which finally suppresses PPARα (the nuclear receptor peroxisome proliferator-activator α) expression in an m^6^A-YTHDF2-dependent manner, which will influence the lipid metabolism 100. Recently, it has been reported that m^6^A modification enriched in and enhanced the expression of lipogenic genes in leptin receptor-deficient *db/db* mice, however, YTHDC2 could attenuate lipid metabolic disorder and hepatic TG accumulation by binding and decreasing the stability of lipogenic gene including sterol regulatory element-binding protein 1c (Srebp-1c), fatty acid synthase (Fasn), stearoyl-CoA desaturase 1 (Scd1) and acetyl-CoA carboxylase 1 (Acc1) 101. m^6^A modification enhances the stability of LINC00958, and subsequently, elevated LINC00958 sponges miR-3619-5p to upregulate hepatoma-derived growth factor (HDGF) expression, thereby facilitating HCC lipogenesis and progression 102. FTO is known to be tightly associated with increased body mass and obesity in humans 103-105. FTO is reported to and positively related to obesity and T2D, and FTO levels were significantly increased in NAFLD group 106,107. These results reveal that m^6^A modification involved in regulating lipogenesis and NAFLD, which is a major risk of HCC.

## Virus infection

Driving factors in hepatocyte transformation and HCC development are chronic inflammation, epigenetic modifications, early neoangiogenesis, senescence, DNA damage, and telomerase reactivation, chromosomal instability 108. Liver cancer usually develops only after decades of HCV infection, and often accompanied by cirrhosis. As a positive-strand RNA virus, most if not all events related to HCV replication are restricted to the cytoplasm. Therefore, HCV infection induced longstanding hepatic inflammation with associated oxidative and the potential DNA damage in the presence of cirrhosis is thus likely to contribute to the development of carcinoma 109,110. HBV contributes to HCC progression through direct and indirect mechanisms. HBV-related HCC, cirrhosis is absent in up to one-third of patient. It has been shown that viral infection can affect m^6^A modification and thus alter m^6^A in special transcripts and influence viral infection 111. m^6^A modification in the 5′ epsilon stem-loop of pgRNA, which mediates HBV life cycle, is necessary for efficient reverse transcription of pgRNA, whereas m^6^A sites in the 3′ epsilon stem-loop lead to destabilization of HBV transcripts 112. This study reveals that m^6^A modification of HBV RNA exerts dual regulatory functions. m^6^A modification negatively regulates HCV life cycle and the production of infectious HCV particles, and m^6^A-binding YTHDF proteins relocalize to lipid droplets, sites of HCV viral assembly, and suppress this stage of viral infection 113. Recently, it has been demonstrated that HBV infection increases METTL3 and subsequently upregulates m^6^A modification and degradation of PTEN, which consequently inhibits IRF-3 nuclear import and activation of PI3K/AKT pathway to facilitate HCC progression by affecting innate immunity 114. Taken together, these studies indicated that m^6^A modification plays a significant role in broader HBV/HCV virus infection, and uncover regulatory strategies to inhibit replication by these established and emerging viral pathogens to prevent HCC development.

## Chromosome Instability

This missegregation of genomic material is defined as Chromosome instability that is associated with poor prognosis of cancer patients including HCC 115,116. HBV can facilitate HCC progression by inducing both chromosome instability by integrating its DNA into the host genome and mutagenesis of various cancer-related genes 117. Furthermore, HBx could directly induce chromosomal instability by affecting the mitotic checkpoints through binding and inactivating p53 and DDB1 118,119. Not only virus DNA, altered cellular signaling pathway could also influence chromosome instability signatures. Weiler and colleagues showed that YAP and its binding partner TEAD4 could induce chromosome instability by interacting with FOXM1 120. After the inhibition of YAP, about four fifth gene of CIN25 and three fifth gene of CIN25 were reduced. Although it hasn't been clarified yet whether m^6^A modification is involved in affecting HCC progression by influencing chromosome instability. Several researchers have shown that YAP mRNA was m^6^A modified in HCC 121, colorectal cancer 122, NSCLC 123, and so on. Those results hint that m^6^A modification may implicated in HCC development through chromosome instability by influencing hepatic virus affection and some genes which need further investigation.

## Neovascularization

Angiogenesis is essential for HCC progression and metastasis. Vasculogenic mimicry (VM) formed by aggressive tumor cells has been found in various types of cancer, including ovarian cancer, colorectal cancer, melanoma and HCC, and associated with poor prognosis in patients 124-126. Pathologic angiogenesis is critical for the growth and malignant dissemination of solid tumors by providing oxygen, nutrients, and routes for metastasis 127. Several studies demonstrated that m^6^A modification could also influence HCC progression through regulating angiogenesis. Qiao *et al.* showed that silencing METTL3 suppresses VM formation through inhibiting YAP1 expression 124. Intratumoral hypoxia is a common signature of human solid cancer including HCC and could regulate the expression of genes that involver in regulating angiogenesis, EMT process, self-renewal of cancer stem cells, chemotherapy- and radiation- therapy resistance extracellular matrix remodeling, cancer stem cell maintenance through hypoxia-induced factor (HIF) 128. Zhong *et al.* demonstrated that hypoxia down-regulated YTHDF2 expression, and forced YTHDF2 expression could suppress HCC carcinogenesis, proliferation and activation of MEK and ERK in HCC cells through directly binding the m^6^A sites on EGFR 3'UTR and subsequently promoting its degradation 129. The activation of EGFR induced by EGF and TGF-α could induce the expression of VEGF which is a predominant stimulator of angiogenesis 130. Those results illustrated that YTHDF2 may be involved in regulating HCC tumorigenesis by regulating angiogenesis through EGFR/VEGF signaling pathway. Hou *et al.* showed YTHDF2 expression was decreased in hypoxia and tumor tissues, and HIF-2α antagonist (PT2385) could restore YTHDF2 expression and inhibit HCC progression 131. Mechanistically, YTHDF2 mediated the decay of m^6^A-modified interleukin 11 (IL11) and serpin family E member 2 (SERPINE2) mRNAs, which were involved in regulating the inflammation-mediated malignancy and disruption of vascular normalization. These studies showed that m^6^A modification might regulating HCC development by modulating neovascularization.

## An altered m^6^A modification in HCC

**Figure 2:**
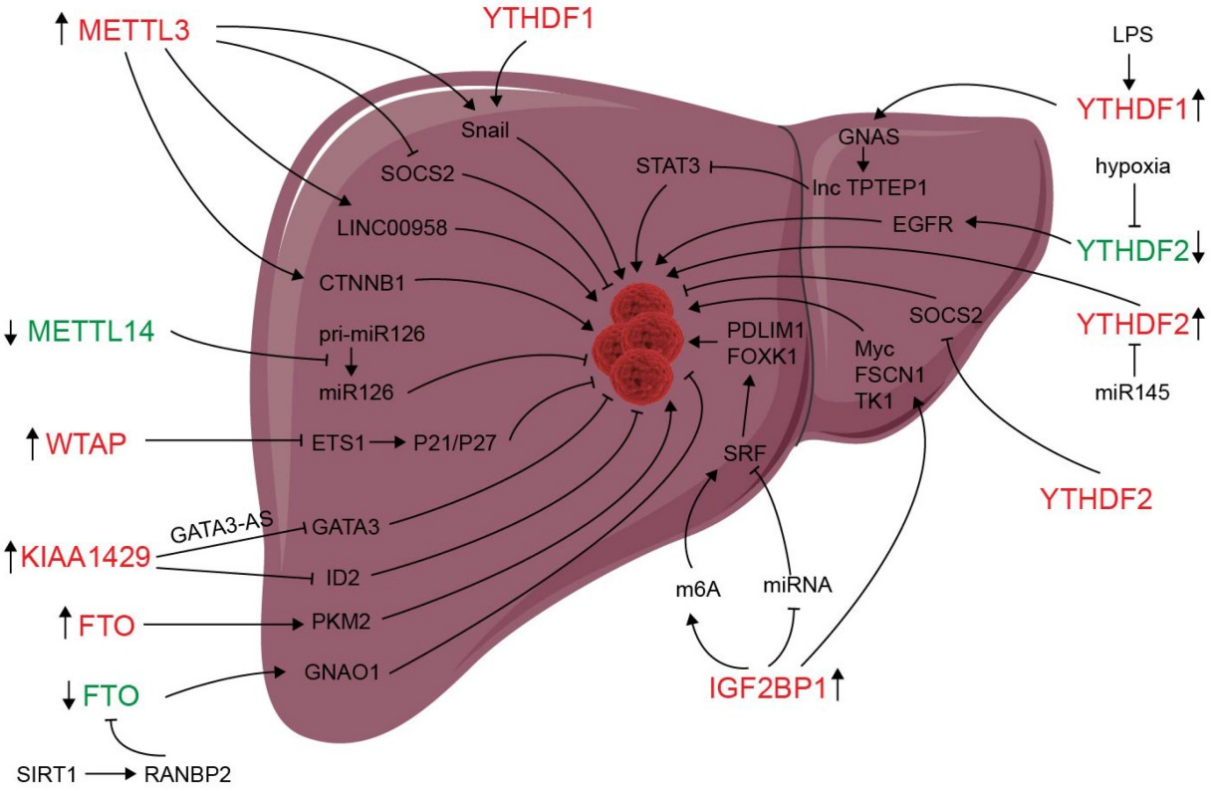
** Deregulation of m6A modifiers in liver cancers.** Modifiers in red indicate an oncogenic role, modifiers in green indicate a tumor-suppressive role.

m^6^A modification and m^6^A regulators have been shown to be dysregulated in liver carcinoma, and m^6^A expression influences liver progression through proliferation, metastasis, inflammation, and vascularization (as showed in **Figure 2**). METTL3 facilitates migration, invasion and EMT of cancer cells by promoting Snail (a key transcription factor of EMT) expression through m^6^A-YTHDF1 pathway 56. SUMOylation of METTL3 was positively associated with high metastasis potential of HCC via controlling Snail mRNA homeostasis in HCC 132. Chen* et al.* showed that METTL3 promotes liver cancer progression by suppressing SOCS2 (suppressor of cytokine signaling 2) via m^6^A-YTHDF2-mediated degradation 55. SOCS2 has been shown to inhibit proliferation, migration, and stemness in various cancers, including oral squamous carcinoma, leukemia, and HCC 54. METTL3-mediated m^6^A modification upregulates LINC00958 expression, and subsequently enhances hepatoma-derived growth factor (HDGF) expression by sponging miR-3619-5p, and consequently promotes HCC lipogenesis and progression 102. METTL3 promoting HCC development by interacting with DGCR8 and promoting the maturation of miR-873-5p in an m^6^A-dependent manner, thereby inhibiting SMG1 expression 133. METTL3 upregulation promotes hepatoblastoma (HB) progression through enhancing CTNNB1 mRNA stability and subsequently activating Wnt/β-catenin pathway 134. WTAP suppresses ETS proto-oncogene 1 (ETS1) in an m^6^A-HuR-dependent manner, which further reverses the inhibition of p21/p27 axis to promote G2/M phase of HCC cells, and consequently promotes HCC progression 83. KIAA1429 upregulation facilitates migration and invasion of HCC by repressing ID2 mRNA in an m^6^A-dependent manner 135. lncRNA-GATA3-AS facilitates KIAA1429 induces m^6^A modification and degradation of GATA binding protein 3 (GATA3) pre-mRNA in an HuR-dependent manner, and consequently promotes HCC growth and metastasis 136. Circ_KIAA1429 (has_circ_0084922) was upregulated and accelerate HCC development by facilitating HCC migration, invasion, and EMT process through enhancing Zeb1 mRNA stability in an m^6^A-YTHDF3 dependent manner 137. However, METTL14 is downregulated in HCC, especially metastasis cancer, can interact with the microprocessor protein DGCR8 and promote pri-miR126 maturation in an m^6^A-dependent manner, and consequently suppresses HCC metastasis 3. In summary, those results reveal that m^6^A “writers” complex involved in regulating HCC progression. However, the function of METTL14 and other m^6^A “writers” is apparently controversial. The reasons for the above conflicting findings might be associated with the heterogeneity of clinical samples. Further investigations are needed to settle these contradictory findings and clarify the role of different m^6^A “writer” in the progression of HCC.

Since its identification as the demethylase of m^6^A, FTO has been reported to play an important role in many cancers including liver cancer 138-141. Li *et al.* reported a correlation between up-regulated FTO in liver cancer and poor prognosis. It was also shown that FTO mediates demethylation of PKM2 mRNA and promotes its translation to promote HCC progression 142. Besides its oncogenic function, FTO also suppresses HCC progression. Liu *et al.* reported that SIRT1 activate RANBP2 that mediates FTO SUMOylation and degradation, and subsequently leads to m^6^A deposition in HCC tumor suppressor guanine nucleotide-binding protein G (o) subunit alpha (GNAO1) and inhibits its mRNA expression, and consequently results in liver cancer progression 139. Recently, a study reveals that FTO plays a protective role in HCC development 143. Hepatocyte-specific depletion of FTO not only impacts HCC initiation phase (increased tumor numbers) but also influences HCC development (increased numbers of larger tumors) by inhibiting Cul4a translation 143. Intrahepatic cholangiocarcinoma (ICC) is the second most common form of primary liver cancer. Rong *et al.* demonstrated that loss of FTO in ICC related to poor prognosis, mechanistically, FTO suppresses the anchorage-independent growth and mobility of ICC cells through demethylation and impairing oncogene TEAD2 mRNA stability 140. These conflicting functions of FTO in HCC and ICC might be associated to heterogeneity of clinical samples and the context-specific m^6^A roles between HCC and ICC. ALKBH5 inhibits HCC cells proliferation and invasion by suppressing IGF2BP1-mediated LY6/PLAUR Domain Containing 1 (LYPD1) RNA stability 144.

The oncogenic or tumor suppressor role of m^6^A readers may be associated with their effect on transcripts processing. Several studies have indicated that chronic inflammation is involved in HCC progression 145. Innate immune cell types, including macrophages, innate lymphoid cells, NK cells, Dendritic cells (DCs), and mucosal-associated invariant T cells (MALTs) are enriched in liver and play essential roles in liver homeostasis 146. Traditionally, macrophages can be classified as M1 (pro-inflammatory), M2 (anti-inflammatory), or M_reg_ (immunosuppressive), and the balance of M1-M2 polarization involved in hepatic inflammation and repair 147. It has been demonstrated that METTL3 facilitates M1 macrophage polarization via the methylation of STAT1 mRNA 148. Ding *et al.* found that LPS used to stimulate the inflammatory response in HCC cells enhances GNAS expression in an m^6^A-YTHDF1-dependent manner 149. Elevated G-protein alpha-subunit (GNAS) expression can promote inflammation-related HCC progression through promoting STAT3 activation by inhibiting lncRNA TPTEP1 interaction with STAT3 (signal transducer and activator of transcription 3). YTHDF2 promotes the liver cancer stem cell phenotype and cancer metastasis by promoting OCT4 expression 150. Hou and colleagues described that hypoxia-inducible factor-2α (HIF-2α) inhibits YTHDF2 expression, resulting in elevated expression of m^6^A-containing interleukin 11 (IL11) and serpin family E member 2 (SERPINE2) mRNAs, which further leads to inflammation and vascular abnormalities in HCC 151. YTHDF2, downregulated by hypoxia, can suppress HCC progression through binding m^6^A sites of EGFR 3'UTR and inducing its degradation, and consequently suppresses cell proliferation, tumor growth, and activation of MEK and ERK in HCC cells 129. However, another research reported that YTHDF2 upregulated and exerts as an oncogene in HCC and can be inhibited by miR-145 152. ICF2BP1/2/3 protein were identified as m^6^A-binding proteins and plays an oncogenic role in HeLa (cervical cancer) and HepG2 (liver cancer) cells via enhancing the stability of MYC, FSCN1, and TK1 mRNAs 35. IGF2BP1 promotes SRF expression in an m^6^A- and miRNA- dependent manner and subsequently promotes SRF downstream target gene including PDLIM7 and FOXK1 translation, consequently PDLIM7 and FOXK1 promote tumor cell growth and enhance cell invasion, and indicate a poor overall survival probability in liver, ovarian and lung cancer 153.

These studies illustrate a complicated role of m^6^A modification and m^6^A regulators in liver carcinogenesis. m^6^A modification and m^6^A regulators involve in regulating cancer cell proliferation, migration, invasion, immunity, vascular abnormalization, and EMT process. However, some of the studies have reported conflicting results on the expression and function of various m^6^A regulators. Most of MTCs upregulated and promote HCC progression, however, METTL14 is decreased and could suppressed liver cancer cell metastasis. The role of FTO and YTHDF2 are double-edged sword. When they down-regulated in carcinoma tissues, they may play a tumor suppressive role. However, if they up-regulated, they may be an oncogenic factor. The expression of FTO and YTHDF2 can be influenced by tumor heterogeneity, hypoxia, and post-translational modification. All the discrepant results of the above studies uncover a complicated role of m^6^A modification and m^6^A regulators in human HCC. Further investigation and effort will be required to reconcile these paradoxical results. Altered expression, function, and mechanism of m^6^A regulators in liver carcinoma are listed in **Table 1**.

## Prognosis and therapeutic potency of targeting altered m^6^A regulators

Since m^6^A modification levels and m^6^A regulators play essential roles in regulating the progression of liver disease, targeting abnormal expressed m^6^A regulators may serve as a biomarker and potential therapeutic targets. Several studies showed that liver carcinogenesis is related to the altered expression of m^6^A regulators 3,129,135. Bioinformation analysis in several studies reveal that METTL3 and YTHDF1 are both significantly upregulated and are associated with poor prognosis in HCC patients 53,154,155. METTL3, VIRMA, and hnRNPC showed higher expression in tumor samples, while METTL14, ZC3H13, FTO, ALKBH5, YTHDF2, YTHDC1, and YTHDC2 showed higher expression in normal tissue samples 53,152,156,157. High expression level of METTL14 is associated with a better prognosis in HCC patients 156. YTHDF2 expression is closely associated with HCC malignance, and can be inhibited by miR-145 152. YTHDF2, YTHDF1, METTL3, KIAA1429, and ZC3H13 could be a prognostic signature of malignant HCC patients 52,157-159. The expression of two genes (hnRNPA2B1 and RBM15) are closely related to the TNM stage and metastasis risk and can act as a prognostic indicator for HBV-related HCC patients in the early TNM stage 160. In conclusion, METTL3, YTHDF1 are elevated and METTL14 is down-regulated in HCC and can be prognosis biomarker of HCC.

m^6^A levels in circulating tumor cells (CTCs) can be detected by liquid chromatography-electrospray ionization-tandem mass spectrometry (LC-ESI-MS/MS). The authors showed that m^6^A modification levels in CTCs was significantly elevated compared to the whole blood cells and m^6^A levels in CTCs might be a non-invasive approach for cancer diagnosis 161. Therefore, the altered expression of m^6^A regulators may be a biomarker for prognostic prediction in HCC patients.

Several studies demonstrated that dysregulation of m^6^A regulators associated with drug resistance. Taketo and colleagues showed that METTL3-depleted pancreatic cancer cells showed higher sensitivity to anticancer reagents such as 5-fluorouracil, gemcitabine, cisplatin and irradiation 162. METTL3 knockdown in NSCLC inhibits tumor growth, metastasis and DDP resistance 163. METTL3 induces the production of p53 R273H mutant protein that results in acquired multidrug resistance in colon cancer cells 164. Elevated METTL3 expression is implicated in glioma stem-like cell maintenance and radioresistance 165. The upregulation of FTO enhances chemo-radiotherapy resistance in cervical cancer 166. Depletion of METTL3 under hypoxia significantly enhances sorafenib-resistant of HCC by decreasing the stability of FOXO3, and overexpression of FOXO3 restores m^6^A-dependent sorafenib sensitivity 167. These results highlight the therapeutic value of targeting m^6^A modification and m^6^A regulators in drug-resistant tumors.

m^6^A regulators can be targeted by inhibitors. The nature product rhein is the first identified FTO inhibitor that exerts good inhibitory activity of FTO demethylation and led to increases of m^6^A modification levels 168,169. However, it is not an FTO selective inhibitor, as it can also target ALKBH5 170. Entacapone, an FDA approved drug used to treat Parkinson's disease, has also be identified as FTO inhibitor that elicited the effects of FTO on gluconeogenesis in the liver and thermogenesis in adipose tissues in mice by acting on an FTO-FOXO1 regulatory axis 171. Meclofenamic acid (MA), a non-steroidal, anti-inflammatory drug, is to be identified as highly selective inhibitor of FTO 30. Most recently, Su* et al.* reported two small-molecule inhibitors of FTO that can suppress leukemia stem/initiating cell maintenance and immune evasion by suppressing expression of immune checkpoint genes, especially LILRB4 172. Administration of PT2358, an antagonist HIF-2α, restores YTHDF2 expression which attenuates tumorous inflammation and angiogenesis, thus inhibiting HCC progression 151.

The m^6^A modification in liver can be influenced by gut microbiota, which can further alter lipid metabolism and insulin signaling 173. Lu *et al.* reported that curcumin can affect METTL3, METTL14, ALKBH5, FTO, and YTHDF2 expression and subsequently increase m^6^A modification to inhibit LPS-induced liver injury and lipid metabolism disorders in piglets through inhibiting SREBP-1c (sterol regulatory element binding proteins) and SCD-1 (stearoyl-CoA desaturase 1) expression in an m^6^A dependent manner 174. Those studies reveal that altered m^6^A modification involved in regulating liver cancer cell metabolism, proliferation, invasion, and immunity, and is associated with clinical prognosis and progression, indicating that altered expression of m^6^A regulators may serve as potential biomarkers and therapeutic targets. However, more studies are required to determine the function of m^6^A modification on liver carcinogenesis and the potential as therapeutic targets.

## Challenges and Perspectives

m^6^A is the most abundant internal modification which randomly exists on almost all types of RNAs, including mRNA, lncRNA, circRNA, miRNA, rRNA and snoRNA. Numerous studies have shown that m^6^A modification plays an essential role in physiological and pathological processes including cell differentiation, meiosis, sex determination, cancer progression, circadian rhythm, neuronal function, and chromatin state. m^6^A modification is induced by MTC consisting of METTL3, METTL14, WTAP, VIRMA, ZC3H13, HAKAI and RBM15/RBM15B, in which METTL3 is the only catalytic subunit. m^6^A in structural RNAs and U6 snRNAs can be methylated by METTL16, whereas METTL5 mediates m^6^A modification of 18 S rRNA and facilitates ZCCHC4 as the 28 S rRNA modification enzyme. m^6^A_m_ modification is induced by PCIF1 and METTL4 mediates m^6^A_m_ modification in internal U2 snRNAs. m^6^A deposition is removed by eraser proteins (FTO and ALKBH5). As the first identified m^6^A demethylase, FTO can not only demethylate internal m^6^A and cap m^6^A_m_ but also mediates demethylation of m^6^A and m^6^A_m_ in snRNAs and m^1^A in tRNAs.

In recent years, global m^6^A modification has been well described well in different disease contexts by using NGS. m^6^A deposition is not randomly distributed in transcripts but collectively deposited in 3'UTR and CDS regions, especially the stop codon. m^6^A deposition sites can be influenced by writers, erasers, histone modification, transcription factors, virus infection, chemical carcinogens, carRNAs, and environmental stimuli. The global m^6^A abundance can be detected by MeRIP-seq or m^6^A-seq. However, this approach has some limitations; it needs more initial RNA material; the m^6^A-antibody cross talks with m^6^A_m_ and it cannot accurately position on m^6^A sites. Another technique uses m^6^A-sensitive RNA-endoribonuclease, which can cleave RNA at unmethylated ACA motifs and is used to detect m^6^A distribution (m^6^A-REF-seq or MAZTER-seq). However, this method is not useful for m^6^A sites in other sequence contexts. Mapping m^6^A sites precisely is a critical step for understanding its function and regulatory pathways. In this context, DART-seq has substantially improved the transcriptome-wide m^6^A level detection and provides an efficient detection method for limiting-amounts of RNA samples.

In many disease types, global m^6^A abundance, as well as m^6^A regulators, are altered. Because of the simultaneous alteration of m^6^A writers and erasers, the global m^6^A level changes may not be informative in carcinoma. Some investigators demonstrated that increased m^6^A modification in liver carcinoma is associated with poor prognosis. However, other groups demonstrated that global m^6^A levels decrease in HCC tissues especially metastatic tumors. Therefore, the mechanism of m^6^A regulation in liver carcinoma progression needs further investigation. Recent studies also revealed that m^6^A modification affects ncRNAs processing such as maturation, cellular location, and function. Further investigations are required to understand the molecular details of these progresses affecting liver cancer progression.

## Conclusions

m^6^A modification is a new layer of post-transcription regulation of gene expression. The dysregulated expression of m^6^A regulators involved in human carcinogenesis has been demonstrated in various cancer types, including HCC. Altered m^6^A regulators expression will influence target gene expression by modulating the mRNA stability and translation. m^6^A modification also plays an essential role in mediating cancer cells response to immunotherapy, radiotherapy, and chemotherapy. However, the functional role of m^6^A in HCC are paradoxical, further studies are needed to illustrates the heterogeneity and complexity of m^6^A modification and m^6^A regulators in HCC carcinogenesis. Given the pivotal role of m^6^A modification and regulation in many cancers, targeting dysregulated m^6^A regulators may be a therapeutic option in cancer. Therefore, a better understanding of m^6^A may improve cancer therapy in the future.

## Figures and Tables

**Table 1:** Roles of m^6^A regulators in liver carcinoma

Proteins	Cancer	Role	Function	Mechanism	Reference
METTL3	HCC	Oncogene	Regulating EMT progression	Promoting Snail expression	56, 132
	HCC	Oncogene	Promoting HCC cell proliferation and migration	Regulating SOCS2 expression	55
	HCC	Oncogene	Promoting HCC lipogenesis and progression	m^6^A modification enhances LINC00958 RNA stability and expression that sponged miR-3619-5p to upregulate HDGF expression	102
	HCC	Oncogene	Promoting the development of HCC	Promoting the maturation of miR-873-5p and thereby inhibiting SMG1 expression	133
	HB	Oncogene	Promoting proliferation of HB	Regulating Wnt/β-catenin pathway activation through regulating CTNNB1 expression	134
METTL14	HCC	Suppressor	Inhibiting the migration and invasiveness	Regulating pre-miR-126 maturation through interacting with DGCR8	3
WTAP	HCC	Oncogene	WTAP modulated the G2/M phase of HCC cells	Suppressing ETS1 expression in m^6^A-HuR dependent manner	83
KIAA1429	HCC	Oncogene	Enhancing proliferation, migration, and invasion of HepG2 cells	Inhibiting ID2 expression	135
	HCC	Oncogene	Promoting proliferation and metastasis of HCC cells	Leading to the degradation of GATA3 pre-mRNA	136
FTO	HCC	Oncogene	Promoting proliferation and *in vivo* tumor growth	Promote PKM2 expression	142
	HCC	Suppressor	SIRT1 activates RANBP2 that further mediates FTO SUMOylation and degradation	Decreased FTO leads to hypo m^6^A modification of GNAO1 and reduced its RNA stability	139
	HCC	Suppressor	FTO exerts protective role in the initiation of HCC	Inhibiting Cul4a translation	143
	ICC	Suppressor	FTO suppresses the anchorage-independent growth and mobility of ICC cells	Impairing oncogene TEAD2 mRNA stability	140
ALKBH5	HCC	Suppressor	ALKBH5 inhibits the proliferation and invasion capabilities of HCC cells *in vitro* and* in vivo*	Reducing the stability of LYPD1 mediated by m^6^A reader IGF2BP1	144
YTHDF1	HCC	Oncogene	Regulating EMT progression	Promote METTL3 target gene snail translation	56
	HCC	Oncogene	Promoting LPS-induced HCC cell growth and invasion	Promoting GNAS translation	149
YTHDF2	HCC	Oncogene	Promoting HCC proliferation and migration	Interacting with METTL3 to regulating SOCS2 expression	55
	HCC	Oncogene	Promoting the liver cancer stem cell phenotype and cancer metastasis	Promoting OCT4 expression	150
	HCC	Suppressor	YTHDF2 silencing provokes inflammation, vascular reconstruction and metastatic progression of HCC	Mediating decay of IL11 and SERPINE2	151
	HCC	Suppressor	Suppressing HCC cell proliferation, growth and induces apoptosis	Destabilizing of EGFR mRNA	129
YTHDF3	HCC	Oncogene	Facilitating HCC migration, invasion, and EMT process	Enhancing Zeb1 mRNA stability	137
IGF2BP1	HCC	Oncogene	Promoting tumor cells proliferation colony formation ability and cell migration/invasion	Enhancing the stability of MYC, FSCN1, and TK1	35
	HCC	Oncogene	Promoting tumor cells proliferation and invasion	Enhancing SRF mRNA stability in an m^6^A-dependent manner	153

## Abbreviations

m^6^A: N^6^-methyladenosine
HCC: Hepatocellular Carcinoma
Ψ: pseudouridine
m^1^A: N^1^-methyladenosine
m^6^A_m_: N^6^, 2-O-dimethyladenosine
ncRNAs: non-coding RNAs
CDS: coding sequences
3'UTRs: 3'untranslated regions
MTC: m^6^A methylase complex
IGF2BPs: insulin-like growth factor 2 mRNA-binding proteins)
NAFLD: non-alcoholic fatty liver disease
HPLC-MS/MS: liquid chromatography coupled to tandem mass spectrometry
PA-m^6^A-seq: photo-crosslinking-assisted m^6^A sequencing strategy
m^6^A-CLIP-seq: UV cross-linked m^6^A antibody-bound RNA sample
a^6^A: N^6^-allyladenosine
hm^6^A: N^6^-hydroxymethyladenosine
dm^6^A: N^6^-dithiolsitolmethyladenosine
m^7^G: 7-methylguanosine
carRNAs: chromosome-associated regulatory RNAs
FMRP: fragile X mental retardation protein
CSC: cigarette smoke condensate
PM: particulate matter
HMPV: human metapneumovirus
dCas9: dead Case9
NASH: nonalcoholic steatohepatitis
HFD: high-fat diet
T2D: Type 2 diabetes
Fasn: fatty acid synthase
PPARα: the nuclear receptor peroxisome proliferator-activator α
Scd1: stearoyl-CoA desaturase 1
Acc1: acetyl-CoA carboxylase 1
HDGF: hepatoma-derived growth factor
HBV: Hepatitis B virus
HB: hepatoblastoma
ETS1: ETS proto-oncogene 1
GATA3: 3′ UTR of GATA binding protein 3
GNAO1: guanine nucleotide-binding protein G (o) subunit alpha
ICC: Intrahepatic Cholangiocarcinoma
DCs: dendritic cells
MALTs: mucosal-associated invariant T cells
GNAS: G-protein alpha-subunit
HIF-2α: hypoxia-inducible factor-2α
IL11: Interleukin 11
SERPINE2: serpin family E member 2
CTCs: circulating tumor cells
LC-ESI-MS/MS: liquid chromatography-electrospray ionization-tandem mass spectrometry
MA: Meclofenamic acid
SREBP-1c: sterol regulatory element-binding protein 1c
SCD-1: stearoyl-CoA desaturase 1
